# Genome-wide meta-analysis in lichen sclerosus identifies 14 genomic risk loci

**DOI:** 10.1093/bjd/ljag088

**Published:** 2026-03-19

**Authors:** Nick Dand, Tuntas Rayinda, Eeva Sliz, Laurent F Thomas, Jake R Saklatvala, Sheila M McSweeney, Chuin Ying Ung, Evangelos Christou, Fiona Lewis, Johannes Kettunen, Laura Huilaja, Ben M Brumpton, Kristian Hveem, Mari Løset, Kaisa Tasanen, John A McGrath, Michael A Simpson, Christos Tziotzios

**Affiliations:** Department of Medical and Molecular Genetics, School of Basic and Medical Biosciences, Faculty of Life Sciences and Medicine, King’s College London, London, UK; St John’s Institute of Dermatology, Faculty of Life Sciences and Medicine, King’s College London, London, UK; Department of Dermatology and Venereology, Faculty of Medicine, Public Health, and Nursing, Universitas Gadjah Mada, Yogyakarta, Indonesia; Systems Epidemiology, Research Unit of Population Health, Faculty of Medicine, University of Oulu, Biocenter Oulu, Oulu, Finland; Medical Research Center Oulu, Oulu University Hospital and University of Oulu, Oulu, Finland; HUNT Center for Molecular and Clinical Epidemiology, Department of Public Health and Nursing, NTNU – Norwegian University of Science and Technology, Trondheim, Norway; Department of Medical and Molecular Genetics, School of Basic and Medical Biosciences, Faculty of Life Sciences and Medicine, King’s College London, London, UK; St John’s Institute of Dermatology, Faculty of Life Sciences and Medicine, King’s College London, London, UK; St John’s Institute of Dermatology, Faculty of Life Sciences and Medicine, King’s College London, London, UK; St John’s Institute of Dermatology, Faculty of Life Sciences and Medicine, King’s College London, London, UK; St John’s Institute of Dermatology, Faculty of Life Sciences and Medicine, King’s College London, London, UK; Systems Epidemiology, Research Unit of Population Health, Faculty of Medicine, University of Oulu, Biocenter Oulu, Oulu, Finland; Medical Research Center Oulu, Oulu University Hospital and University of Oulu, Oulu, Finland; Department of Dermatology, Medical Research Center Oulu, Oulu University Hospital and Research Unit of Clinical Medicine University of Oulu, Oulu, Finland; HUNT Center for Molecular and Clinical Epidemiology, Department of Public Health and Nursing, NTNU – Norwegian University of Science and Technology, Trondheim, Norway; HUNT Center for Molecular and Clinical Epidemiology, Department of Public Health and Nursing, NTNU – Norwegian University of Science and Technology, Trondheim, Norway; HUNT Center for Molecular and Clinical Epidemiology, Department of Public Health and Nursing, NTNU – Norwegian University of Science and Technology, Trondheim, Norway; Department of Dermatology, Medical Research Center Oulu, Oulu University Hospital and Research Unit of Clinical Medicine University of Oulu, Oulu, Finland; St John’s Institute of Dermatology, Faculty of Life Sciences and Medicine, King’s College London, London, UK; Department of Medical and Molecular Genetics, School of Basic and Medical Biosciences, Faculty of Life Sciences and Medicine, King’s College London, London, UK; St John’s Institute of Dermatology, Faculty of Life Sciences and Medicine, King’s College London, London, UK

## Abstract

**Background:**

Lichen sclerosus (LS) is a common and highly debilitating chronic inflammatory dermatosis that primarily affects genital skin in both sexes. Despite the utility of large genetic studies to reveal pathogenic mechanisms and suggest novel therapeutic targets, the genetic basis of LS remains largely unstudied.

**Objectives:**

To identify genomic loci at which common genetic variation influences LS susceptibility and establish associated pathogenic mechanisms.

**Methods:**

Sex-stratified genome-wide association studies of genital LS were performed in three European biobanks [UK Biobank, the Trøndelag Health Study (HUNT) and FinnGen]. Cases of LS were primarily identified via linked electronic health records from primary and/or secondary care. In total, 6681 female patients with LS and 407 255 female control individuals were included along with 970 male patients with LS and 331 484 male control individuals. Genome-wide association studies were combined through fixed-effect meta-analysis. Further analyses to identify putative causal variants and genes were performed, including statistical fine-mapping, functional annotation and assessment of colocalization with gene expression quantitative trait loci (eQTLs).

**Results:**

The major histocompatibility complex class II allele HLA-DRB1*12:01 was strongly associated with LS susceptibility in females [odds ratio (OR) 2.54, 95% confidence interval (CI) 2.28–2.83; *P* = 8.3 × 10^–63^] and in males (OR 2.30, 95% CI 1.73–3.07; *P* = 1.3 × 10^–8^). We found genome-wide significant associations (*P* < 5.0 × 10^–8^) at another 12 loci in females, including a potentially causal protein-altering variant in *IGFLR1* [rs140952221; OR 0.71, 95% CI 0.67–0.74 (*P* = 2.1 × 10^–11^)]. Colocalization with established eQTLs in skin and immune tissues highlighted dysregulated expression of *CD247*, *IL2RB* and *ALDH2* among potential LS pathomechanisms. Evidence was found for shared genetic effects between the sexes, albeit with diluted effect sizes in males (β = 0.42, 95% CI 0.21–0.64; *P* = 9.7 × 10^–5^). One further risk locus, at chromosome 5p13.2 was revealed by combined-sex meta-analysis.

**Conclusions:**

This study provides new insight into the pathogenesis of LS by identifying common genetic variation contributing to disease risk and implicating immune–inflammatory and metabolic pathways as potential future drug targets.

## Teaser

Lichen sclerosus (LS) is a common and highly debilitating chronic inflammatory dermatosis, but its genetic basis remains poorly understood. We conducted sex-stratified genome-wide association studies in three European biobanks (6681 cases of female LS and 970 male cases) and identified HLA-DRB1*12:01 as a major risk allele, 12 additional female-specific loci and a shared locus on chromosome 5p13.2. Colocalization with expression quantitative trait loci highlighted dysregulated immune and metabolic genes, providing new insights into LS pathogenesis and potential therapeutic targets.

Linked Article: Simmonds and Petukhova. *Br J Dermatol* 2026; **195**:10–12.

What is already known about this topic?

- Lichen sclerosus (LS) is a common and highly debilitating chronic inflammatory dermatosis that primarily affects genital skin in female and male individuals.
- There are shared and distinct clinical features of LS between the sexes.
- Although familial segregation is suggestive of genetic contribution, and small-scale studies have inconsistently implicated major histocompatibility (MHC) class I and II alleles, there has been no systematic research into the genetic aetiopathogenesis of LS.

What does this study add?

- A genome-wide association meta-analysis, including 6681 female and 970 male patients with LS, identified the class II MHC allele HLA-DRB1*12:01 as strongly associated with susceptibility in both males and females.
- Common genetic variation at a further 12 genomic loci were associated with LS in females, and an additional locus in both sexes.

What is the translational message?

- The findings identify key LS genetic risk factors and provide novel insight into immune-mediated pathogenic mechanisms that may offer new opportunities for risk stratification and therapeutic targeting.

Lichen sclerosus (LS) is a common and highly debilitating chronic inflammatory dermatosis that primarily affects the genital skin in both sexes.^1^ Histologically, lesions are similar between female and male individuals, and there are shared pathomechanisms between LS and other fibrosing conditions.^2^ Malignant transformation to squamo-proliferative neoplasia is seen both in female and male LS. However, there are also sex-specific clinical features: LS is more prevalent in women, who also suffer a higher burden of autoimmune comorbidities than men, and substantially higher rates of extragenital cutaneous involvement, which can resemble scleroderma and morphoea.^2^ Surgical treatment can be curative in men; it is only undertaken in women presenting with severe functional sequelae or neoplastic transformation, and is associated with high recurrence rates, often due to koebnerization.^3^ These factors suggest that shared and sex-specific mechanisms underpin LS pathogenesis.

Although familial segregation is suggestive of genetic contribution, and small-scale studies have inconsistently implicated major histocompatibility complex (MHC) class I and class II alleles, there has been no systematic research into the genetic aetiopathogenesis of LS.^2^ Here, we identify genomic loci at which genetic variation influences susceptibility to LS by undertaking a series of case–control genome-wide association studies (GWAS) in European ancestry cohorts.

## Patients and methods

We identified cases of female and male LS in three independent European data resources [UK Biobank, the Trøndelag Health Study (HUNT) and FinnGen]. Case ascertainment was based on primary and secondary care electronic health records, using International Classification of Diseases (ICD) 10th Revision code L90.0, suitably mapped terms in other coding systems or self-report in UK Biobank (Appendix S1; see Supporting Information). Control participants were individuals with no record of LS.

Sex-stratified and combined GWAS were performed in each dataset using genome-wide imputed data, with appropriate measures taken to control for population structure (see Appendix S1). We undertook sex-specific and combined standard error-weighted fixed-effect meta-analyses across up to 27 684 790 biallelic variants (Table S1; see Supporting Information). Our primary analysis focused on female LS (6681 patients and 407 255 healthy control participants), and we performed comparative analysis (regression of estimated effects at lead variants) with male LS (970 patients and 331 484 healthy control participants) and a sex-combined meta-analysis. To fine-map association signals in the MHC region, a similar meta-analysis design was used for classical human leucocyte antigen (HLA) alleles imputed in the UK Biobank and FinnGen datasets. At female and combined non-MHC susceptibility loci, putative underlying causal variants were identified using the approximate Bayes factor method implemented in coloc version 5.2.3,^4^ and annotated with predicted protein-coding effects using ANNOVAR.^5^ For candidate variants with a posterior probability of being causal > 0.1, we checked for previously reported associations with gene expression in blood, skin and immune tissues using the eQTL Catalogue (see Appendix S1),^6^ and with other diseases or traits using GWAS Catalog.^7^ Formal colocalization analysis was performed for selected traits using coloc. Full details of the methodology are included in Appendix S1.

## Results

### Genetic associations with female lichen sclerosus

**Figure 1:**
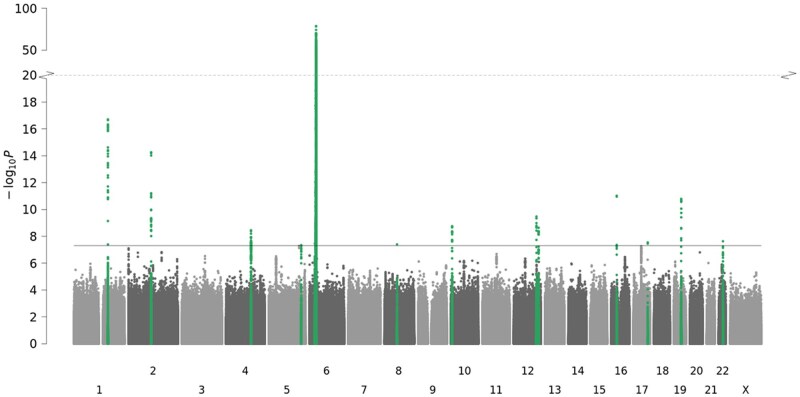
Manhattan plot demonstrating evidence for association with lichen sclerosus (LS) in a meta-analysis of 6681 European women with LS and 407 255 healthy control participants. The *x*-axis shows the genomic position and the *y*-axis the −log_10_(*P*-value) of association (two-sided *Z*-test, unadjusted for multiple tests). Green points indicate regions associated with LS susceptibility at genome-wide significance (*P* = 5 × 10^−8^). The solid horizontal line indicates the genome-wide significance threshold and the dotted horizontal line, the *y*-axis break at 10^−20^. Chromosomes (labelled 1–22 and X) are alternately shaded for clarity.

In females, we found genetic variants with genome-wide significant evidence of association with LS susceptibility at 13 genomic loci [Figure 1, Table 1; Table S2 (see Supporting Information)]. Linkage disequilibrium score regression estimated that common genetic variants explain 6.7% of variance in the liability to female LS [assuming a disease prevalence of 1% (Table S3; see Supporting Information)].

**Table 1:** Genome-wide significant associations with lichen sclerosus in females

rsID	Chromosome	Position (hg19)	Band	Effect allele	Noneffect allele	Effect allele frequency	OR (95% CI)	*P*-value	Direction	Candidate gene(s)
rs2949661	1	167 424 924	1q24.2	T	C	0.338	0.85 (0.82–0.88)	2.0 × 10^–17^	–––	*CD247*
rs6732565	2	111 607 832	2q13	A	G	0.607	1.15 (1.11–1.20)	5.5 × 10^–15^	+++	*LINC01123*
rs539870576	4	123 036 810	4q27	T	C	0.081	1.21 (1.14–1.29)	3.6 × 10^–9^	++?	–
rs6887783	5	159 922 209	5q34	T	G	0.669	1.11 (1.07–1.15)	4.6 × 10^–8^	+++	–
rs112457525	6	32 415 429	6p21.32	A	G	0.023	2.43 (2.21–2.66)	2.3 × 10^–79^	+++	*HLA-DRB1*
rs79376089	8	61 841 979	8q12.2	A	G	0.057	1.22 (1.14–1.31)	4.0 × 10^–8^	+++	–
rs10795791	10	6 108 340	10p15.1	A	G	0.525	0.90 (0.87–0.93)	1.8 × 10^–9^	–––	–
rs4766578	12	111 904 371	12q24.12	A	T	0.537	0.89 (0.86–0.93)	3.3 × 10^–10^	–––	*ALDH2,* *(SH2B3)*
rs7968808	12	122 661 791	12q24.31	T	C	0.440	1.11 (1.07–1.15)	2.3 × 10^–9^	+++	*LRRC43*
rs76715626	16	27 398 042	16p12.1	T	C	0.942	0.72 (0.66–0.79)	9.5 × 10^–12^	?––	–
rs187661678	17	70 036 684	17q24.3	T	C	0.004	5.74 (3.10–10.63)	2.9 × 10^–8^	?++	–
rs200440599	19	36 188 481	19q13.12	A	AAAG	0.051	0.69 (0.62–0.77)	1.6 × 10^–11^	––?	*IGFLR1,* *(ZBTB32)*
rs228955	22	37 532 699	22q12.3	T	C	0.463	0.91 (0.87–0.94)	2.3 × 10^–8^	–––	*IL2RB*

Direction indicates direction of effect in UK Biobank, FinnGen and Trøndelag Health Study (HUNT) studies, respectively. CI, confidence interval; OR, odds ratio.

The strongest evidence for association was found at the 6p21.32 locus within the MHC region [lead variant rs112457525; odds ratio (OR) 2.43, *P* = 2.3 × 10^–79^]. To refine this signal to a causal allele, we assessed the evidence for association with LS of imputed four-digit classical MHC class I and II alleles. The strongest association was found for the class II allele HLA-DRB1*12:01 [OR 2.54, 95% confidence interval (CI) 2.28–2.83; *P* = 8.3 × 10^–63^] (Table S4; see Supporting Information).

### Functional mapping of lichen sclerosus association signals

To investigate possible disease mechanisms at non-MHC susceptibility loci, we conducted statistical and functional fine-mapping at each locus. Approximate Bayesian fine-mapping identified 95% credible sets of 10 or fewer putative causal variants at 6 of the 12 female non-MHC loci (Figure S1, Table S5; see Supporting Information), including a single high-confidence variant at chromosome 8q12.2 [rs79376089, posterior probability (PP) = 0.95]. Of 27 strong candidate variants (PP > 0.1) at these loci, 3 were predicted to be protein-altering (Table S6; see Supporting Information), with the nonsynonymous variant rs140952221 at 19q13.12 strongly predicted to have a deleterious effect on *IGFLR1* [*P*_meta_ = 2.1 × 10^–11^, PP_fine-mapping_ = 0.231, combined annotation dependent depletion (CADD) score 21.1]. Protein-altering variants were also identified in *ZBTB32* at the same locus, and in *SH2B3* at chromosome 12q24.12, albeit with lower predicted likelihood of impairing protein function (CADD scores 13.1 and 13.9, respectively).

We next searched the eQTL Catalogue^6^ for evidence that any of our 27 strong candidate variants are associated with gene expression levels in relevant skin, blood and immune tissues (see Appendix S1), reasoning that shared associations could implicate the regulation of specific genes in LS pathobiology. This approach identified expression quantitative trait loci (eQTLs) for 26 gene–tissue pairs corresponding to nine genes across six female LS loci (Table S7; see Supporting Information), and we formally tested all genes in selected tissues for colocalization between eQTLs and LS susceptibility (Table S8; see Supporting Information).

At 1q24.2, the lead single nucleotide polymorphism (SNP) identified by statistical fine-mapping (rs2949661, PP = 0.239) is an intronic variant within *CD247* for which the minor (T) allele is protective for LS. While the LS signal did not formally colocalize with an eQTL for *CD247* expression in whole blood (PP_coloc_ = 4.3 × 10^–4^), it did colocalize with eQTL data derived from CD4^+^ T cells (PP_coloc_ = 0.912), providing evidence that the same variant that contributes reduced risk of LS at this locus is also associated with increased expression of *CD247*.

At locus 12q24.12, the lead SNP is rs4766578 (PP = 0.379), a known regulatory variant for the aldehyde hydrogenase gene *ALDH2*. Indeed, the LS susceptibility signal colocalized with an eQTL conferring decreased expression of aldehyde hydrogenase in neutrophils (PP_coloc_ = 0.989), indicating a shared causal variant. This finding may reflect the immune cell context in which the regulatory effect is most readily detected, rather than neutrophil-mediated inflammation in LS. The relevance of this finding is unclear and may reflect systemic rather than lesion-confined pathomechanisms that are yet to be elucidated. Evidence that LS risk alleles are associated with increased use of the canonical transcript (ENST00000261733) suggests that there may be transcript-specific effects. This locus has been associated with a wide range of other traits, including immune-mediated and cardiometabolic diseases, such as coronary artery disease and alcohol-induced cardiac dysfunction (Table S9; see Supporting Information). In a skin disease context, we found that the same causal variants that increase LS risk at this locus increase the risk of vitiligo (PP_coloc_ = 0.987).

We also noted that LS susceptibility alleles colocalized with increased expression of *IL2RB* in skin at chr22q12.3 (PP_coloc_ = 0.844), increased expression of *LRRC43* in natural killer cells at chr12q24.31 (PP_coloc_ = 0.979) and decreased expression of *LINC01123* in thyroid at chr2q13 (PP_coloc_ = 0.912). Conversely, we found no evidence for colocalization between LS susceptibility and *IGFLR1* expression at chr19q13.12 (PP_coloc_ = 1.2 × 10^–7^), consistent with the above-described strong candidate deleterious coding variant underlying this association.

### Genetic associations with male lichen sclerosus

Finally, while the substantially smaller number of male LS patients (*n* = 970) limited statistical power for novel variant discovery, we assessed evidence for association with LS at the 13 female lead SNPs (Table S10; see Supporting Information). Genome-wide significant evidence of association was identified within the MHC region and fine-mapping implicated the same class II allele that underlies female LS, HLA-DRB1*12:01, with consistent effect size (OR 2.30, 95% CI 1.73–3.07; *P* = 1.3 × 10^–8^) (see Table S4). Comparison of effect sizes across loci indicated correlation between the sexes, albeit with evidence of effect size dilution in males [inverse-variance weighted regression slope = 0.42, 95% CI 0.21–0.64 (*P* = 9.7 × 10^–5^); excluding MHC lead variant, slope = 0.26, 95% CI 0.03–0.50 (*P* = 0.03)] (Figure S2; see Supporting Information). However, these findings should be interpreted with caution, due to the wide CIs around male effect size estimates.

Given this evidence for potential shared LS genetic architecture between the sexes, we examined sex-combined meta-analysis results, finding one further genome-wide significant LS susceptibility locus at 5p13.2 [lead variant rs1494564; OR 0.91, 95% CI 0.87–0.94 (*P* = 4.7 × 10^–8^)] (Table S11, Figure S3; see Supporting Information). Statistical fine-mapping did not confidently resolve the signal to a causal variant; the 95% credible set included 31 variants (rs1494564 PP = 0.067), but we noted that rs6897932 is a missense variant affecting *IL7R* (PP = 0.043, CADD score 7.3).

## Discussion

This study identifies genome-wide significant (*P* < 5 × 10^−8^) associations with LS at 13 genomic loci in female individuals, and at an additional locus when data are combined with that from male individuals, and thereby characterizes male and female LS as complex traits underpinned by common genetic variation. Moreover, we demonstrate that risk variants at some female loci also contributed to male LS susceptibility, but with a substantially attenuated effect size. Indeed, while not significant genome wide, the strongest evidence for association with male LS outside of the MHC region was found outside of female LS susceptibility loci (Table S12; see Supporting Information). These findings suggest that male and female LS share a genetic background but may also be underpinned by distinct pathomechanisms affected by sex-specific genetic and environmental factors, which may partly explain their distinct and overlapping clinical features.

The strongest association was identified within 6p21.32, which corresponds to HLA-DRB1*12:01, conferring a roughly 2.5-fold higher risk of developing LS in both sexes. This finding aligns with a previous report that linked HLA-DRB1*12:01 to vulvar LS,^8^ and provides robust support that MHC class II molecules, which are known to be involved in antigen presentation in a plethora of autoimmune diseases, is of key significance in male and female LS. *HLA-DRB1* alleles have previously been associated with increased risk of rheumatoid arthritis,^9^ type 1 diabetes mellitus,^10^ multiple sclerosis (MS),^11^ coeliac disease,^12^ myasthenia gravis^13^ and pemphigus,^14^ among other autoimmune traits.^15–17^

At 1q24.2, we implicated *CD247* through colocalization of the LS association with a CD4^+^ T-cell eQTL. *CD247* encodes the T-cell receptor (TCR) T3 zeta chain, a glycoprotein that forms part of the TCR–CD3 complex and plays an important role in several intracellular signal transduction pathways activated by antigen binding. *CD247* has also been associated with systemic sclerosis (SSc),^18^ which shares clinical and histological features with extragenital LS. At 10p15.1, the lead risk variant rs10795791 lies immediately upstream of *IL2RA*, a gene involved in T-cell activation and previously associated with SSc.^19^ This finding further supports shared underlying pathobiology between LS and SSc. Additionally, the association with other fibrotic diseases, such as MS, is supported by our findings at 4q27 and 5p13.2. These loci encompass the genes *IL21* and *IL7R*, respectively, both of which have been previously implicated in MS.^20,21^

Our analysis has identified additional genes implicated in T-cell homeostasis and cutaneous inflammatory response. At 2q13, *LINC01123* emerged as the causal gene candidate by colocalization with eQTL data in thyroid tissue; *LINC01123* plays a crucial role in suppressing CD8^+^ T-cell activation.^22^ At 19q13.12, statistical fine-mapping implicates a protein-altering variant (p.C112Y) in exon 3 of *IGFLR1*, which encodes the insulin growth factor-like receptor (IGFLR). The IGFLR1 protein has similar structure to tumour necrosis factor (TNF) receptor family members and is expressed across immune cell types.^23^ Moreover, human insulin-like growth factor 1 expression is enhanced by TNF-α treatment and has been shown to be upregulated in psoriatic skin samples, suggesting that *IGFLR1* is likely involved in skin inflammation.^23^

The susceptibility locus identified at chromosome 12q24.12 has been previously characterized for its broad pleiotropic effects,^24^ including on vitiligo, a frequent comorbidity of LS.^25^ By colocalization analysis, we robustly implicated regulatory variants at *ALDH2* in the LS disease process. *ALDH2* encodes aldehyde dehydrogenase 2, an enzyme critical to alcohol metabolism. *ALDH2* variants specific to East Asian populations are strongly associated with alcohol intolerance,^26^ and regulatory variants have been implicated in immune and metabolic phenotypes, including pathways related to oxidative stress.^27^ Therefore, it is possible that the association between genetic variation at 12q24.12 and LS risk is mediated, at least in part, through immune regulatory or oxidative stress mechanisms; further studies are warranted to determine whether this effect relates to alcohol intake, residual alcohol metabolites influencing immune function, or other non-alcohol-related pathways.

The present study included women and men of European ancestry and therefore the study of LS in men and women from more diverse backgrounds is crucial to establish the generalizability of the observed associations. Moreover, the male cohort was significantly smaller than the female cohort, limiting statistical power to detect male-specific effects and perform comparative analyses. Further studies in larger male cohorts are necessary. Another limitation of this study is the reliance on ICD-based case definitions for LS, which may be subject to some degree of diagnostic misclassification; however, given the relatively high clinical specificity of LS, the diagnosis of which is usually made on the basis of clinical features alone, any such misclassification is likely to have a small impact on the observed associations.^2^

In summary, we demonstrate that the risk of female and male individuals developing LS is driven by variation at multiple genomic loci, including a primary association with the MHC class II allele HLA-DRB1*12:01. We identify probable disease mechanisms by implicating genes in T-cell response, antigen presentation and fibrosis-related pathways. These observations provide novel insight into observed clinical associations with other fibrotic and inflammatory skin conditions and should direct translational research towards efforts to target the associated pathways. Moreover, the association with genetic variation affecting *ALDH2*, with the established role of its encoded protein in the oxidative pathway of alcohol metabolism, provides direction for further research. Taken together, our findings characterize LS as an MHC class II immune-mediated, genetically complex inflammatory and fibrosing trait. They provide a platform for future functional and genetic epidemiological research into improved prognostication, risk stratification and drug discovery for this debilitating inflammatory disease.

## Supplementary Material

ljag088_Supplementary_Data

## Data Availability

The summary statistics generated from this study can be accessed in the GWAS Catalogue (https://ftp.ebi.ac.uk/pub/databases/gwas/summary_statistics/GCST90824001-GCST90825000/), accession identification GCST90824100 (female), GCST90824101 (male) and GCST90824102 (combined).
