# Supplementary material for: Genome-wide meta-analysis in lichen sclerosus identifies 14 genomic risk loci
Source: Br J Dermatol. 2026 Mar 19;195(1):102–8. doi: 10.1093/bjd/ljag088 (PMC13289594; doi:10.1093/bjd/ljag088)
Supplement: ljag088_Supplementary_Data [file ljag088_supplementary_data.zip › BJD_LS_GWAS_Supporting_Table_Figures_resub_180126.docx]

Supporting Table and Figures

**Genome-wide meta-analysis in lichen sclerosus identifies 14 genomic risk loci**

Nick Dand, Tuntas Rayinda, Eeva Silz, Laurent F Thomas, Jake R Saklatvala, Sheila M McSweeney, Chuin Ying Ung, Evangelos Christou, Fiona Lewis, Johannes Kettunen, Laura Huilaja, Ben M. Brumpton, Kristian Hveem, Mari Løset, Kaisa Tasanen, John A McGrath, Michael A Simpson, Christos Tziotzios

Correspondence: ND (nick.dand@kcl.ac.uk), MAS (michael.simpson@kcl.ac.uk) and CT (christos.tziotzios@kcl.ac.uk)

**Supplementary Tables**

**Table S1** – Final sample and variant numbers included in each GWAS

| **Study** | **Male** | | | **Female** | | | **All** | | |
| --- | --- | --- | --- | --- | --- | --- | --- | --- | --- |
|  | Cases | Controls | Variants tested | Cases | Controls | Variants tested | Cases | Controls | Variants tested |
| FinnGen | 485 | 204,762 | 21,315,091 (20,337,994) | 3,303 | 260,911 | 21,320,476 (20,342,892) | 3,788 | 465,673 | 21,327,267 (20,348,683) |
| HUNT | 195 | 40,601 | 17,690,308 | 771 | 45,278 | 18,046,632 | 966 | 85,879 | 19,807,394 |
| UK Biobank | 290 | 86,121 | 9,614,655 | 2,607 | 101,066 | 9,614,469 | 2,897 | 187,187 | 9,614,694 |
| **Meta-analysis** | **970** | **331,484** | **25,925,086** | **6,681** | **407,255** | **26,192,342** | **7,651** | **738,739** | **27,684,790** |

Counts in brackets for FinnGen are number of variants successfully lifted over to GRCh37.

**Table S2** – Results per contributing study for genome-wide significant associations in the female lichen sclerosus meta-analysis

| **rsID** | **Chr.** | **Position (hg19)** | **Band** | **Effect Allele** | **Non-effect allele** | **UK Biobank** | | | **FinnGen** | | | **HUNT** | | |
| --- | --- | --- | --- | --- | --- | --- | --- | --- | --- | --- | --- | --- | --- | --- |
|  |  |  |  |  |  | **EAF** | **Odds ratio**  **(95% CI)** | **P-value** | **EAF** | **Odds ratio**  **(95% CI)** | **P-value** | **EAF** | **Odds ratio**  **(95% CI)** | **P-value** |
| rs2949661 | 1 | 167,424,924 | 1q24.2 | T | C | 0.405 | 0.84  (0.79-0.88) | 7.8E-10 | 0.259 | 0.86  (0.82-0.92) | 6.2E-07 | 0.380 | 0.84  (0.76-0.93) | 0.001 |
| rs6732565 | 2 | 111,607,832 | 2q13 | A | G | 0.621 | 1.14  (1.07-1.21) | 1.5E-05 | 0.598 | 1.17  (1.11-1.23) | 6.8E-10 | 0.602 | 1.13  (1.02-1.25) | 0.024 |
| rs539870576 | 4 | 123,036,810 | 4q27 | T | C | 0.074 | 1.19  (1.07-1.31) | 0.001 | 0.085 | 1.23  (1.13-1.33) | 1.0E-06 | - | - | - |
| rs6887783 | 5 | 159,922,209 | 5q34 | T | G | 0.662 | 1.11  (1.05-1.18) | 0.001 | 0.675 | 1.10  (1.05-1.16) | 2.1E-04 | 0.667 | 1.12  (1.00-1.25) | 0.041 |
| rs112457525 | 6 | 32,415,429 | 6p21.32 | A | G | 0.015 | 2.06  (1.74-2.43) | 2.6E-14 | 0.026 | 2.58  (2.31-2.88) | 9.2E-63 | 0.016 | 3.76  (1.80-7.85) | 4.2E-04 |
| rs79376089 | 8 | 61,841,979 | 8q12.2 | A | G | 0.045 | 1.24  (1.10-1.40) | 0.001 | 0.065 | 1.19  (1.09-1.31) | 2.5E-04 | 0.047 | 1.38  (1.06-1.79) | 0.017 |
| rs10795791 | 10 | 6,108,340 | 10p15.1 | A | G | 0.588 | 0.92  (0.87-0.97) | 0.004 | 0.472 | 0.87  (0.83-0.91) | 1.3E-08 | 0.549 | 0.97  (0.87-1.07) | 0.547 |
| rs4766578 | 12 | 111,904,371 | 12q24.12 | A | T | 0.502 | 0.88  (0.83-0.93) | 9.0E-06 | 0.571 | 0.92  (0.88-0.97) | 0.001 | 0.509 | 0.83  (0.75-0.92) | 4.4E-04 |
| rs7968808 | 12 | 122,661,791 | 12q24.31 | T | C | 0.459 | 1.13  (1.07-1.20) | 9.2E-06 | 0.423 | 1.09  (1.04-1.14) | 0.001 | 0.450 | 1.14  (1.03-1.26) | 0.014 |
| rs76715626 | 16 | 27,398,042 | 16p12.1 | T | C | - | - | - | 0.940 | 0.72  (0.66-0.79) | 1.4E-11 | 0.992 | 0.77  (0.43-1.40) | 0.390 |
| rs187661678 | 17 | 70,036,684 | 17q24.3 | T | C | - | - | - | 0.000 | 5.78  (2.08-16.04) | 0.001 | 0.007 | 5.71  (2.63-12.39) | 1.0E-05 |
| rs200440599 | 19 | 36,188,481 | 19q13.12 | A | AAAG | 0.018 | 0.59  (0.45-0.77) | 3.4E-05 | 0.056 | 0.71  (0.64-0.80) | 1.3E-08 | - | - | - |
| rs228955 | 22 | 37,532,699 | 22q12.3 | T | C | 0.422 | 0.91  (0.86-0.97) | 0.001 | 0.496 | 0.90  (0.85-0.94) | 1.1E-05 | 0.449 | 0.92  (0.83-1.03) | 0.138 |

Chr., chromosome; EAF, effect allele frequency; CI, confidence interval

**Table S3** – Estimated variation in LS risk explained by common genetic variants

| **Assumed prevalence** | **Females** | **Males** | **Sex-combined** |
| --- | --- | --- | --- |
| 0.5% | 0.0575 (0.0113) | 0.0015 (0.0516) | 0.0506 (0.0097) |
| 1% | 0.067 (0.0131) | 0.0018 (0.0601) | 0.059 (0.0113) |
| 2% | 0.0796 (0.0156) | 0.0021 (0.0714) | 0.0701 (0.0134) |

Liability-scale heritability explained by common variants estimated using LD score regression

**Table S4** – Sex-specific association results for classical HLA alleles

Based on meta-analysis of UK Biobank and FinnGen datasets. Top five alleles based on marginal p-value are reported per sex.

| **Rank** | **Allele** | **Freq.** | **Odds ratio**  **(95% CI)** | **P-value** |
| --- | --- | --- | --- | --- |
| Female |  |  |  |  |
| 1 | HLA-DRB1*12:01 | 0.022 | 2.54  (2.28-2.83) | 8.3E-63 |
| 2 | HLA-DQA1*05:05 | 0.070 | 1.82  (1.63-2.03) | 1.7E-27 |
| 3 | HLA-DQB1*03:01 | 0.148 | 1.37  (1.29-1.45) | 1.3E-26 |
| 4 | HLA-DPB1*02:01 | 0.126 | 0.66  (0.61-0.72) | 1.1E-23 |
| 5 | HLA-DRB4*99:01 | 0.648 | 0.79  (0.74-0.84) | 3.1E-14 |
| Male |  |  |  |  |
| 1 | HLA-DRB1*12:01 | 0.022 | 2.30  (1.73-3.07) | 1.3E-8 |
| 2 | HLA-DPB1*04:01 | 0.418 | 1.33  (1.18-1.50) | 4.6E-6 |
| 3 | HLA-DPB1*02:01 | 0.130 | 0.64  (0.52-0.80) | 5.5E-5 |
| 4 | HLA-C*15:05 | 0.000 | 43.09  (6.89-269.65) | 5.8E-5 |
| 5 | HLA-DQB1*02:02 | 0.066 | 1.44  (1.15-1.81) | 1.3E-3 |

**Table S5** – Statistical fine-mapping at non-MHC loci

| **Locus** | **Highest PP variant** | **Position (hg19)** | **PP** | **95% credible set size** |
| --- | --- | --- | --- | --- |
| *Female* | | | | |
| 1q24.2 | rs2949661 | 167,424,924 | 0.239 | 10 |
| 2q13 | rs6732565 | 111,607,832 | 0.434 | 3 |
| 4q27 | rs539870576 | 123,036,810 | 0.050 | 224* |
| 5q34 | rs5010836 | 159,919,166 | 0.166 | 11 |
| 8q12.2 | rs79376089 | 61,841,979 | 0.950 | 1 |
| 10p15.1 | rs10795791 | 6,108,340 | 0.142 | 14 |
| 12q24.12 | rs4766578 | 111,904,371 | 0.379 | 6 |
| 12q24.31 | rs7968808 | 122,661,791 | 0.159 | 12 |
| 16p12.1 | rs76715626 | 27,398,042 | 0.544 | 2 |
| 17q24.3 | rs568116062 | 70,286,004 | 0.033 | 9,172^†^ |
| 19q13.12 | rs200440599 | 36,188,481 | 0.269 | 6 |
| 22q12.3 | rs228955 | 37,532,699 | 0.345 | 11 |
| *Combined sex* | | | | |
| 5p13.2 | rs1494564 | 35,851,831 | 0.067 | 31 |

PP, posterior probability (of being the true causal variant underlying the signal)

* 4q27 locus fine-mapped over a window of ±1000 kb due to extended LD pattern

^†^ The Bayesian model at the 17q24.3 locus attributed a posterior probability of 0.100 to “null” (no association). Therefore the 95% credible set defaults to all variants in the region.

**Table S6** – Protein-altering variants in fine-mapping credible sets

| **Locus** | **Position**  **(hg19)** | **Ref** | **Alt** | **Gene** | **Consequence** | **Transcript effects** | **CADD** | **gnomAD AAF FIN** | **gnomAD AAF NFE** | **PP**  **(rank)** |
| --- | --- | --- | --- | --- | --- | --- | --- | --- | --- | --- |
| *Female* | | | | | | | | | | |
| 4q27 | 123,276,961 | G | A | *KIAA1109* | synonymous SNV | KIAA1109:NM_015312.3:exon81:c.G14316A:p.L4772L | . | 0.085 | 0.081 | 0.002 (=206/224) |
| 12q24.12 | 111,884,608 | T | C | *SH2B3* | nonsynonymous SNV | SH2B3:NM_001291424.1:exon2:c.T178C:p.W60R SH2B3:NM_005475.2:exon3:c.T784C:p.W262R | 13.9 | 0.601 | 0.523 | 0.112 (3/6) |
| 19q13.12 | 36,206,203 | T | A | *ZBTB32* | nonsynonymous SNV | ZBTB32:NM_014383.3:exon3:c.T675A:p.S225R | 13.1 | 0.056 | 0.025 | 0.210 (3/6) |
| 19q13.12 | 36,231,288 | C | T | *IGFLR1* | nonsynonymous SNV | IGFLR1:NM_001346006.2:exon3:c.G335A:p.C112Y IGFLR1:NM_024660.4:exon3:c.G335A:p.C112Y | 21.1 | 0.056 | 0.025 | 0.231 (2/6) |
| 22q12.3 | 37,531,436 | G | A | *IL2RB* | synonymous SNV | IL2RB:NM_000878.5:exon8:c.C750T:p.G250G IL2RB:NM_001346222.1:exon8:c.C750T:p.G250G IL2RB:NM_001346223.2:exon8:c.C750T:p.G250G | . | 0.516 | 0.439 | 0.037 (8/11) |
| *Combined sex* | | | | | | | | | | |
| 5p13.2 | 35,874,575 | C | T | *IL7R* | nonsynonymous SNV | IL7R:NM_002185.5:exon6:c.C731T:p.T244I | 7.3 | 0.335 | 0.267 | 0.043 (11/31) |

Ref, reference allele; alt, alternative allele; AAF FIN, gnomAD alternative allele frequency amongst Finnish genomes; AAF NFE, gnomAD alternative allele frequency amongst non-Finnish European genomes; PP (rank), posterior probability estimated by statistical fine-mapping and rank within 95% credible set; SNV, single nucleotide variant.

**Table S7** – Summary of eQTL Catalogue associations at strong candidate lichen sclerosus variants

| **Locus** | **Tissue label** | **Gene** | **N_datasets_** |
| --- | --- | --- | --- |
| 1q24.2 | blood | *CD247* | 6 |
|  | CD4+ T cell | *CD247* | 7 |
|  | CD4+ TCM cell | *CD247* | 1 |
|  | CD8+ T cell | *CD247* | 4 |
|  | NK cell | *CD247* | 3 |
|  | T cell | *CD247* | 3 |
|  | Tfh cell | *CD247* | 3 |
|  | Th1 cell | *CD247* | 1 |
|  | Th17 cell | *CD247* | 4 |
|  | Th2 cell | *CD247* | 1 |
|  | Treg memory | *CD247* | 7 |
|  | Treg naive | *CD247* | 2 |
| 2q13 | thyroid | *LINC01123* | 1 |
| 12q24.12 | neutrophil | *ALDH2* | 2 |
|  | blood | *MAPKAPK5-AS1* | 1 |
| 12q24.31 | NK cell | *LRRC43* | 2 |
|  | blood | *MLXIP* | 2 |
|  | thyroid | *MLXIP* | 4 |
|  | blood | *VPS33A* | 2 |
|  | skin | *VPS33A* | 2 |
|  | skin (suprapubic) | *VPS33A* | 1 |
|  | thyroid | *VPS33A* | 1 |
| 19q13.12 | plasma | *IGFLR1* | 1 |
| 22q12.3 | skin | *IL2RB* | 3 |
|  | skin (suprapubic) | *IL2RB* | 2 |
|  | Treg memory | *IL2RB* | 1 |

Table summarizes the number of datasets included in the eQTL Catalogue in which one of our strong candidate lichen sclerosus variants (statistical fine-mapping posterior probability >0.1) has genome-wide significant evidence of association (P<5.0×10^-8^) with expression of each gene.

**Table S8** – Colocalization analysis with selected eQTL associations

| **Locus** | **Gene** | **Coloc analysis** | **Tissue** | **Molecular trait ID** | **Study** | **Evidence for colocalization** | | | | | **Implicated gene?** |
| --- | --- | --- | --- | --- | --- | --- | --- | --- | --- | --- | --- |
|  |  |  |  |  |  | **PP.H0** | **PP.H1** | **PP.H2** | **PP.H3** | **PP.H4** |  |
|  |  |  |  |  |  |  |  |  |  |  |  |
| 1q24.2 | *CD247* | Attempt 1 | Blood | ENSG00000198821 | Lepik_2017 | 3.3E-204 | 4.8E-193 | 7.0E-12 | **1.000** | 4.3E-04 | Yes: *CD247* |
|  |  | Attempt 2 | CD4+ T cell | ENSG00000198821 | OneK1K | 5.5E-27 | 7.9E-16 | 8.1E-14 | 0.011 | **0.989** |  |
|  |  |  |  |  |  |  |  |  |  |  |  |
| 2q13 | *LINC01123* | Attempt 1 | Thyroid | ENSG00000204588 | GTEx | 9.7E-11 | 0.032 | 1.7E-10 | 0.056 | **0.912** | Yes: *LINC01123* |
|  |  |  |  |  |  |  |  |  |  |  |  |
| 12q24.12 | *MAPKAPK5-AS1* | Attempt 1 * | Blood | ENSG00000234608.9_ 12_111840134_111840332 | Lepik_2017 | 3.1E-34 | 2.5E-30 | 1.3E-04 | **1.000** | 2.5E-05 | Yes: *ALDH2* |
|  | *ALDH2* | Attempt 1 | Neutrophil | ENSG00000111275 | BLUEPRINT | 9.3E-09 | 7.4E-05 | 1.5E-06 | 0.011 | **0.989** |  |
|  |  |  |  |  |  |  |  |  |  |  |  |
| 12q24.31 | *MLXIP* | Attempt 1 | Thyroid | ENSG00000175727_12/  122141083/122141691/  clu_20241_+ | GTEx | 2.2E-69 | 5.6E-66 | 4.0E-04 | **1.000** | 1.4E-05 | Yes: *LRRC43* |
|  |  | Attempt 2 | Blood | ENSG00000175727_12/  122141083/122141408/  clu_12766_+_blood_chr12 | GTEx | 4.7E-22 | 1.2E-18 | 4.0E-04 | **1.000** | 1.7E-05 |  |
|  | *VPS33A* | Attempt 1 | Blood | ENST00000544349 | Lepik_2017 | 7.7E-24 | 1.9E-20 | 4.0E-04 | **1.000** | 2.1E-05 |  |
|  |  | Attempt 2 | Sun-exposed skin | ENST00000267199 | GTEx | 2.2E-08 | 5.4E-05 | 4.0E-04 | **1.000** | 1.5E-05 |  |
|  | *LRRC43* | Attempt 1 | NK-cell | ILMN_2133125 | Gilchrist_ 2021 | 1.4E-09 | 3.5E-06 | 8.8E-06 | 0.021 | **0.979** |  |
|  |  |  |  |  |  |  |  |  |  |  |  |
| 19q13.12 | *IGFLR1* | Attempt 1 * | Plasma | IGFLR1.7244.16.3..1 | Sun_2018 | 8.3E-148 | 1.2E-142 | 7.0E-06 | **1.000** | 1.2E-07 | No |
|  |  |  |  |  |  |  |  |  |  |  |  |
| 22q12.3 | *IL2RB* | Attempt 1 | Skin | ENSG00000100385 | TwinsUK | 9.2E-58 | 1.1E-55 | 1.3E-03 | 0.154 | **0.844** | Yes: *IL2RB* |

* For these genes only a single study with a candidate eQTL was identified.

Molecular traits as listed in eQTL Catalogue.

Evidence for colocalization is summarised as posterior probability (PP) supporting each of five competing hypotheses (Giambartolomei et al, 2014):

H_0_: neither trait has a genetic association in the region

H_1_: only trait 1 has a genetic association in the region

H_2_: only trait 2 has a genetic association in the region

H_3_: both traits are associated, but with different causal variants

H_4_: both traits are associated and share a single causal variant

**Table S9** – Associations reported in GWAS Catalog for strong candidate lichen sclerosus variants

| **Locus** | **Variant** | **rsID** | **N_traits_** | **Traits (alphabetical)** |
| --- | --- | --- | --- | --- |
| 1q24.2 | 1:167424924_C_T | rs2949661 | 18 | CD3 on activated & secreting CD4 regulatory T cell; CD3 on activated CD4 regulatory T cell; CD3 on CD39+ activated CD4 regulatory T cell; CD3 on CD39+ CD4+ T cell; CD3 on CD39+ secreting CD4 regulatory T cell; CD3 on CD4 regulatory T cell; CD3 on CD45RA- CD4+ T cell; CD3 on CD45RA+ CD4+ T cell; CD3 on Central Memory CD4+ T cell; CD3 on Naive CD4+ T cell; CD3 on secreting CD4 regulatory T cell; Eosinophil counts; Hypothyroidism; Medication use (adrenergics, inhalants); Medication use (glucocorticoids); Neutrophil count; White blood cell count |
| 2q13 | 2:111601478_A_G | rs11123201 | 2 | White blood cell count |
|  | 2:111607832_A_G | rs6732565 | 2 | Rheumatoid arthritis; White blood cell count |
|  | 2:111616141_C_T | rs1533299 | 2 | Eosinophil percentage of white cells; White blood cell count |
| 10p15.1 | 10:6106266_C_T | rs7072793 | 1 | Hypothyroidism |
|  | 10:6106638_C_T | rs7096384 | 2 | Hypothyroidism; ICD10 L43: Lichen planus |
|  | 10:6108340_A_G | rs10795791 | 7 | Eosinophil counts; Eosinophil percentage of white cells; Rheumatoid arthritis; Rheumatoid arthritis (ACPA-positive); Type 1 diabetes |
| 12q24.12 | 12:111884608_C_T | rs3184504 | 230 | Acetate levels; Alanine aminotransferase levels; Annual healthcare cost; Apolipoprotein A1 levels; Appendicular lean mass; Aspartate aminotransferase levels; Autoimmune thyroid disease; Basophil count; Beta-2 microglubulin plasma levels; Birth weight; Body mass index; C-C motif chemokine 3 levels; C-X-C motif chemokine 10 levels; C-X-C motif chemokine 11 levels; C-X-C motif chemokine 9 levels; Cardiovascular disease; CD4+ T cell Absolute Count; Celiac disease; Central Memory CD4+ T cell Absolute Count; Cholesterol esters in large LDL; Cholesterol esters in medium LDL; Cholesterol esters in small LDL; Cholesteryl esters to total lipids ratio in large LDL; Cholesteryl esters to total lipids ratio in medium LDL; Cholesteryl esters to total lipids ratio in small LDL; Chronic inflammatory diseases (ankylosing spondylitis, Crohn's disease, psoriasis, primary sclerosing cholangitis, ulcerative colitis) (pleiotropy); Colorectal cancer; Colorectal or endometrial cancer; Concentration of IDL particles; Concentration of large LDL particles; Concentration of medium LDL particles; Concentration of small LDL particles; Coronary artery disease; Coronary artery disease or plasminogen activator inhibitor 1 levels (pleiotropy); Coronary artery disease or tissue plasminogen activator levels (pleiotropy); Cryptic phenotype that captures autosomal dominant polycystic kidney disease severity; Cystatin C plasma levels; Diastolic blood pressure; Diastolic blood pressure (cigarette smoking interaction); Diastolic blood pressure x depressive symptoms interaction (2df test); Endometrial cancer; Endometrial cancer (endometrioid histology); Eosinophil counts; Eosinophil side scatter distribution width; Esterified cholesterol levels; Estimated glomerular filtration rate (creatinine, cystatin c); Estimated glomerular filtration rate (cystatin c); Fibrinogen levels; Fibroblast growth factor-binding protein 1 levels; Free cholesterol in IDL; Free cholesterol in large LDL; Free cholesterol in medium LDL; Glaucoma (multi-trait analysis); Granulocyte count; Hashimoto thyroiditis; HDL cholesterol levels; Heart failure (multivariate analysis); Height; Hematocrit; Hemoglobin; Hemoglobin concentration; Hemoglobin levels; High density lipoprotein cholesterol levels; High light scatter reticulocyte count; High light scatter reticulocyte percentage of red cells; Hip circumference adjusted for BMI; Hypertension (confirmatory factor analysis Factor 12); Hypothyroidism; Hypothyroidism or rheumatoid arthritis (pleiotropy); IDP dMRI TBSS OD Posterior corona radiata R; IgA levels; Immature fraction of reticulocytes; Inflammatory bowel disease; Inflammatory markers and poor diet (confirmatory factor analysis Factor 30); Interleukin-12 subunit beta levels; Ischemic stroke; Ischemic stroke or factor VII levels (pleiotropy); Ischemic stroke or factor VIII levels (pleiotropy); Ischemic stroke or factor XI levels (pleiotropy); Ischemic stroke or fibrinogen levels (pleiotropy); Ischemic stroke or plasminogen activator inhibitor 1 levels (pleiotropy); Ischemic stroke or tissue plasminogen activator levels (pleiotropy); Ischemic stroke or von Willebrand factor levels (pleiotropy); Kynurenine levels; Latent autoimmune diabetes vs. type 2 diabetes; Left ventricular end-diastolic volume; Leukocyte telomere length or COVID-19 infection (MTAG); Liver enzyme levels (alanine transaminase); Low affinity immunoglobulin gamma Fc region receptor III-B levels; Low density lipoprotein cholesterol levels; Lymphocyte count; Medication use (calcium channel blockers); Medication use (thyroid preparations); Metabolite levels (kynurenine; cmh_kynurenine; Kynurenine); Monocyte count; Multiple sclerosis; Myeloid white cell count; Myeloproliferative neoplasms (MTAG); Natural killer cell receptor 2B4 levels; Neutrophil count; Non-HDL cholesterol levels; NT-3 growth factor receptor levels; Offspring birth weight; Parental longevity (father's age at death); Phospholipids in IDL; Phospholipids in large LDL; Phospholipids in medium LDL; Phospholipids in small LDL; Platelet count; Plateletcrit; Primary sclerosing cholangitis; Primary sclerosing cholangitis (MTAG); Quinolinate levels; Red blood cell count; Red blood cell traits; Reticulocyte count; Reticulocyte fraction of red cells; Retinal venular width; Rheumatoid arthritis; Rheumatoid arthritis (rheumatoid factor and/or anti-cyclic citrullinated peptide seropositive); Semaphorin-4D levels; Serum levels of protein CXCL11; Serum total cholesterol levels; Stroke; Sum basophil neutrophil counts; Sum neutrophil eosinophil counts; Systolic blood pressure; Systolic blood pressure (cigarette smoking interaction); T cell Absolute Count; T-cell surface glycoprotein CD5 levels; Thyroid stimulating hormone levels; Tonsillectomy; Total cholesterol in HDL3; Total cholesterol in large LDL; Total cholesterol in medium LDL; Total Cholesterol in small LDL; Total cholesterol levels; Total cholesterol levels in LDL; Total cholesterol to total lipids ratio in large LDL; Total cholesterol to total lipids ratio in medium LDL; Total cholesterol to total lipids ratio in small LDL; Total lipid levels in medium LDL; Total lipids in IDL; Total lipids in large LDL; Total lipids in small LDL; Tumor necrosis factor levels; Type 1 diabetes; Vascular cell adhesion protein 1 levels (VCAM1.2967.8.1); Vitamin D-binding protein levels; Vitamin D-binding protein levels (adjusted for GC haplotype); White blood cell count; Whole body fat free mass (UKB data field 23101); Whole brain restricted directional diffusion (multivariate analysis); X-12100 levels |
|  | 12:111904371_A_T | rs4766578 | 60 | Aging; Apolipoprotein B levels; Arthritis (juvenile idiopathic); Birth weight; Cholesterol levels in large LDL; Cholesterol levels in medium LDL; Cholesterol levels in small LDL; Cholesterol to total lipids ratio in medium LDL; Cholesteryl ester levels in large LDL; Cholesteryl ester levels in LDL; Clinical LDL cholesterol levels; Concentration of LDL particles; Coronary artery disease; Current medication use (UKB data field 20003); Fibrinogen levels or factor VII levels or factor XI levels or tissue plasminogen activator levels (pleiotropy); Fibrinogen levels or tissue plasminogen activator levels (pleiotropy); Free cholesterol in IDL (UKB data field 23528); Free cholesterol in large HDL; Free cholesterol in large LDL (UKB data field 23535); Free cholesterol levels in large LDL; Free cholesterol levels in LDL; Free cholesterol levels in medium LDL; Free cholesterol levels in small LDL; Free cholesterol to total lipids in medium VLDL percentage (UKB data field 23597); Free cholesterol to total lipids ratio in small HDL; HDL cholesterol; High light scatter reticulocyte count; High light scatter reticulocyte percentage of red cells; Immature fraction of reticulocytes; Ischemic heart disease (PheCode 411); LDL cholesterol levels; Linoleic acid levels; Low fluorescent percentage of reticulocytes; Medium fluorescent percentage of reticulocytes; Phospholipid levels in large LDL; Phospholipid levels in LDL; Phospholipid levels in small LDL; Polyunsaturated fatty acid levels; Reaction time; Reticulocyte fraction of red cells; Right ventricular end diastolic volume; Right ventricular stroke volume; Smoking initiation; Smoking initiation (ever regular vs never regular); Smoking status (ever vs never smokers); Telomere length (principal component 1); Total cholesterol levels (UKB data field 23400); Total cholesterol minus HDL-C levels; Total esterified cholesterol levels (UKB data field 23415); Total free cholesterol levels (UKB data field 23419); Total lipid levels in large LDL; Total lipid levels in LDL; Total lipid levels in lipoprotein particles; Total lipid levels in small LDL; Total omega-6 fatty acid levels; Tumor necrosis factor receptor 2 levels; Vitiligo |
|  | 12:111910219_A_G | rs10774625 | 41 | Asthma (childhood onset); Body mass index and coronary artery disease (pairwise); Body mass index and HDL-C (pairwise); Body mass index and systole blood pressure (pairwise); Cholesteryl ester levels in HDL; Cholesteryl esters to total lipids ratio in large HDL; Chronic elevation of alanine aminotransferase (cALT) levels; Coronary artery disease; Coronary artery disease (myocardial infarction, percutaneous transluminal coronary angioplasty, coronary artery bypass grafting, angina or chromic ischemic heart disease); EGF-like repeat and discoidin I-like domain-containing protein 3 levels; Estimated glomerular filtration rate; Externalizing behaviour (multivariate analysis); Fibrinogen levels or plasminogen activator inhibitor 1 levels (pleiotropy); Free cholesterol levels in HDL; Free cholesterol to total lipids ratio in IDL; Free cholesterol to total lipids ratio in medium HDL; Free cholesterol to total lipids ratio in very small VLDL; Glycated hemoglobin levels; HDL cholesterol levels; Hypertension; Hypothyroidism; Large artery stroke (MTAG); Left ventricle diastolic internal dimension; Lifetime smoking; Matrilin-2 levels; Mean spheric corpuscular volume; Mitral valve prolapse; Myocardial infarction; Pain (pleiotropy); Parental longevity (father's age at death or father's attained age); Protein kinase C-binding protein NELL2 levels; Retinal vascular caliber; Rheumatoid arthritis or type 1 diabetes; Smoking initiation; Systemic lupus erythematosus; Urate levels |
| 12q24.31 | 12:122661791_C_T | rs7968808 | 1 | Eczema |
| 16p12.1 | 16:27395662_C_CT | rs201121732 | 2 | Ulcerative colitis |
|  | 16:27398042_C_T | rs76715626 | 1 | Hypothyroidism |

Table includes traits for which a p-value <5.0×10-8 is reported in GWAS Catalog for “strong candidate” LS variants (statistical fine-mapping posterior probability of being causal >0.1) [accessed 15^th^ October 2024].

**Table S10** – Lookup in male meta-analysis of female genome-wide significant associations with lichen sclerosus

| **rsID** | **Chromosome** | **Position (hg19)** | **Band** | **Effect Allele** | **Non-effect allele** | **Effect allele frequency** | **Odds ratio**  **(95% CI)** | **P-value** | **Direction** |
| --- | --- | --- | --- | --- | --- | --- | --- | --- | --- |
| rs2949661 | 1 | 167,424,924 | 1q24.2 | T | C | 0.332 | 0.95  (0.86-1.05) | 0.288 | --- |
| rs6732565 | 2 | 111,607,832 | 2q13 | A | G | 0.606 | 1.09  (0.99-1.19) | 0.080 | -++ |
| rs539870576 | 4 | 123,036,810 | 4q27 | T | C | 0.081 | 1.10  (0.92-1.33) | 0.288 | ++? |
| rs6887783 | 5 | 159,922,209 | 5q34 | T | G | 0.668 | 1.06  (0.96-1.16) | 0.263 | +++ |
| rs112457525 | 6 | 32,415,429 | 6p21.32 | A | G | 0.022 | 1.84  (1.42-2.37) | 3.0×10^-6^ | +++ |
| rs79376089 | 8 | 61,841,979 | 8q12.2 | A | G | 0.057 | 1.09  (0.89-1.33) | 0.406 | -++ |
| rs10795791 | 10 | 6,108,340 | 10p15.1 | A | G | 0.522 | 0.90  (0.82-0.98) | 0.020 | --- |
| rs4766578 | 12 | 111,904,371 | 12q24.12 | A | T | 0.537 | 1.06  (0.97-1.16) | 0.194 | -++ |
| rs7968808 | 12 | 122,661,791 | 12q24.31 | T | C | 0.441 | 0.96  (0.88-1.05) | 0.379 | +-- |
| rs76715626 | 16 | 27,398,042 | 16p12.1 | T | C | 0.943 | 0.82  (0.63-1.07) | 0.150 | ?-- |
| rs187661678 | 17 | 70,036,684 | 17q24.3 | T | C | 0.007 | 1.44  (0.43-4.82) | 0.554 | ?-+ |
| rs200440599 | 19 | 36,188,481 | 19q13.12 | A | AAAG | 0.049 | 1.02  (0.78-1.31) | 0.905 | -+? |
| rs228955 | 22 | 37,532,699 | 22q12.3 | T | C | 0.463 | 1.00  (0.91-1.10) | 0.987 | -++ |

CI, confidence interval. Direction indicates direction of effect in UK Biobank, FinnGen and HUNT studies, respectively.

**Table S11** – Genome-wide significant associations with lichen sclerosus in combined-sex meta-analysis

**A** – Genome-wide significant associations

| **rsID** | **Chromosome** | **Position (hg19)** | **Band** | **Effect Allele** | **Non-effect allele** | **Effect allele frequency** | **Odds ratio**  **(95% CI)** | **P-value** | **Direction** | **Female GWS locus?** |
| --- | --- | --- | --- | --- | --- | --- | --- | --- | --- | --- |
| rs2949661 | 1 | 167,424,924 | 1q24.2 | T | C | 0.337 | 0.86  (0.83-0.89) | 1.5×10^-16^ | --- | Yes |
| rs6732565 | 2 | 111,607,832 | 2q13 | A | G | 0.608 | 1.15  (1.11-1.18) | 1.5×10^-15^ | +++ | Yes |
| rs539870576 | 4 | 123,036,810 | 4q27 | T | C | 0.081 | 1.20  (1.13-1.27) | 2.9×10^-9^ | ++? | Yes |
| rs1494564 | 5 | 35,851,831 | 5p13.2 | T | C | 0.314 | 0.91  (0.87-0.94) | 4.7×10^-8^ | --- | No |
| rs12661806 | 6 | 32,583,540 | 6p21.32 | A | T | 0.977 | 0.42  (0.38-0.46) | 1.7×10^-83^ | --? | Yes |
| rs10795791 | 10 | 6,108,340 | 10p15.1 | A | G | 0.525 | 0.90  (0.87-0.93) | 1.2×10^-10^ | --- | Yes |
| rs76715626 | 16 | 27,398,042 | 16p12.1 | T | C | 0.942 | 0.74  (0.68-0.81) | 7.5×10^-12^ | ?-- | Yes |
| rs140952221 | 19 | 36,231,288 | 19q13.12 | T | C | 0.047 | 0.73  (0.67-0.81) | 1.0×10^-10^ | --- | Yes |

CI, confidence interval; GWS, genome-wide significant. Direction indicates direction of effect in UK Biobank, FinnGen and HUNT studies, respectively.

**B** – Results per contributing study for the 5p13.2 association observed in the combined-sex meta-analysis

| **rsID** | **Chromosome** | **Position (hg19)** | **Study** | **Effect Allele** | **Non-effect allele** | **Effect allele frequency** | **Odds ratio**  **(95% CI)** | **P-value** |
| --- | --- | --- | --- | --- | --- | --- | --- | --- |
| rs1494564 | 5 | 35,851,831 | UK Biobank | T | C | 0.294 | 0.91  (0.85-0.96) | 1.1×10^-3^ |
|  |  |  | FinnGen | T | C | 0.333 | 0.90  (0.86-0.95) | 3.8×10^-5^ |
|  |  |  | HUNT | T | C | 0.287 | 0.92  (0.83-1.02) | 0.115 |

CI, confidence interval.

**Table S12** – Suggestive significant associations with lichen sclerosus in male meta-analysis

| **rsID** | **Chromosome** | **Position (hg19)** | **Band** | **Effect Allele** | **Non-effect allele** | **Effect allele frequency** | **Odds ratio**  **(95% CI)** | **P-value** | **Direction** | **Female**  **GWS locus?** |
| --- | --- | --- | --- | --- | --- | --- | --- | --- | --- | --- |
| rs7590736 | 2 | 37,622,756 | 2p22.2 | T | G | 0.989 | 0.40  (0.28-0.57) | 7.2E-07 | --- | No |
| **rs1042121** | **6** | **33,048,542** | **6p21.32** | **T** | **C** | **0.499** | **0.76**  **(0.69-0.83)** | **3.8E-09** | **---** | **Yes** |
| rs440094 | 14 | 77,703,143 | 14q24.3 | T | C | 0.920 | 0.69  (0.59-0.80) | 7.7E-07 | --- | No |
| rs80249197 | 19 | 57,423,158 | 19q13.43 | A | G | 0.041 | 1.81  (1.44-2.27) | 3.5E-07 | +++ | No |

Includes variants achieving suggestive significance (P<1.0×10^-6^), having minor allele frequency >0.01 and tested in 2+ contributing studies.

CI, confidence interval; GWS, genome-wide significant. Direction indicates direction of effect in UK Biobank, FinnGen and HUNT studies, respectively.

* Variant achieves GWS p-value but not robustly replicated across studies (FinnGen: OR=11.0 [95% CI 4.8-25.6], P=2.6×10^-8^; HUNT: OR=1.15 [95%CI 0.01-189.7], P=0.956)

**Supplementary Figures**

**Figure S1 – Statistical fine-mapping at non-MHC loci**

Points represent individual variants. x-axis, chromosome position; y-axis, negative log_10_ p-value of association from meta-analysis; red points, variants included in 95% credible sets. Note the 4q27 locus is fine-mapped over a window of ±1000 kb due to extended LD pattern, and the Bayesian model at the 17q24.3 locus attributed a posterior probability of 0.100 to “null” (no association). Therefore the 95% credible set defaults to all variants in the region.

**
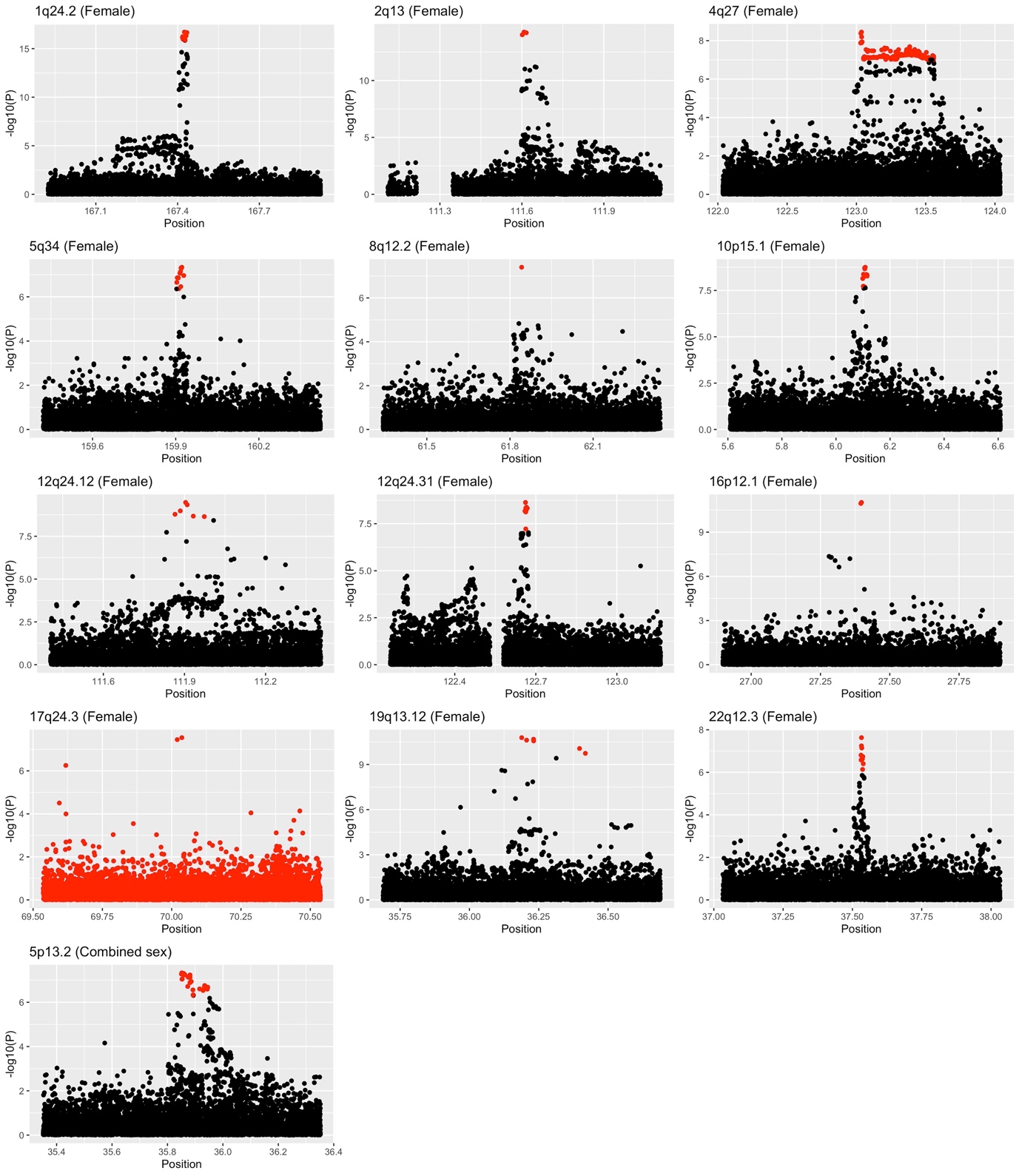
**

**Figure S2** – Comparison of estimated effect sizes in males and females for female lichen sclerosus susceptibility variants

| **A** | **B** |
| --- | --- |
| **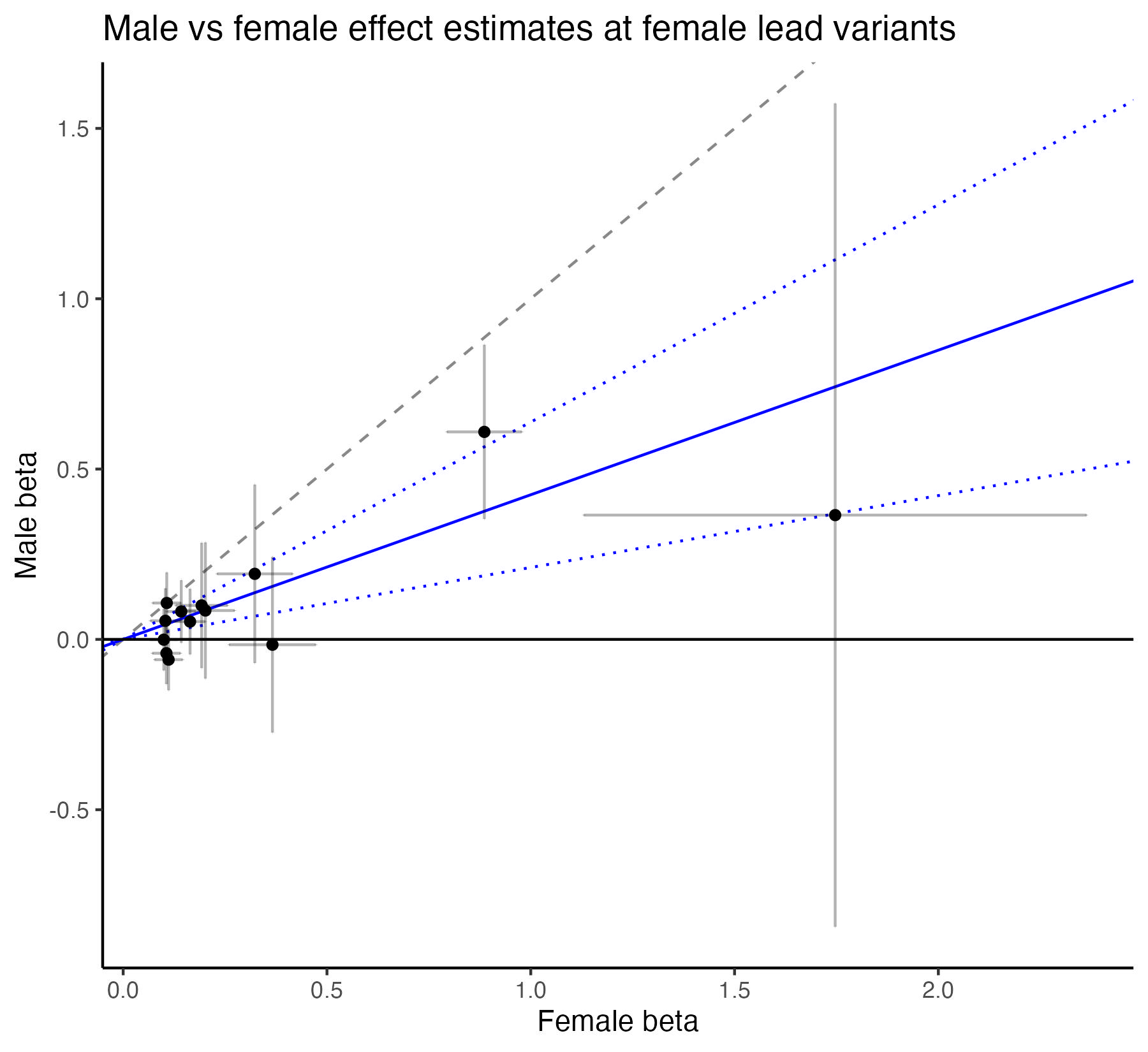** | **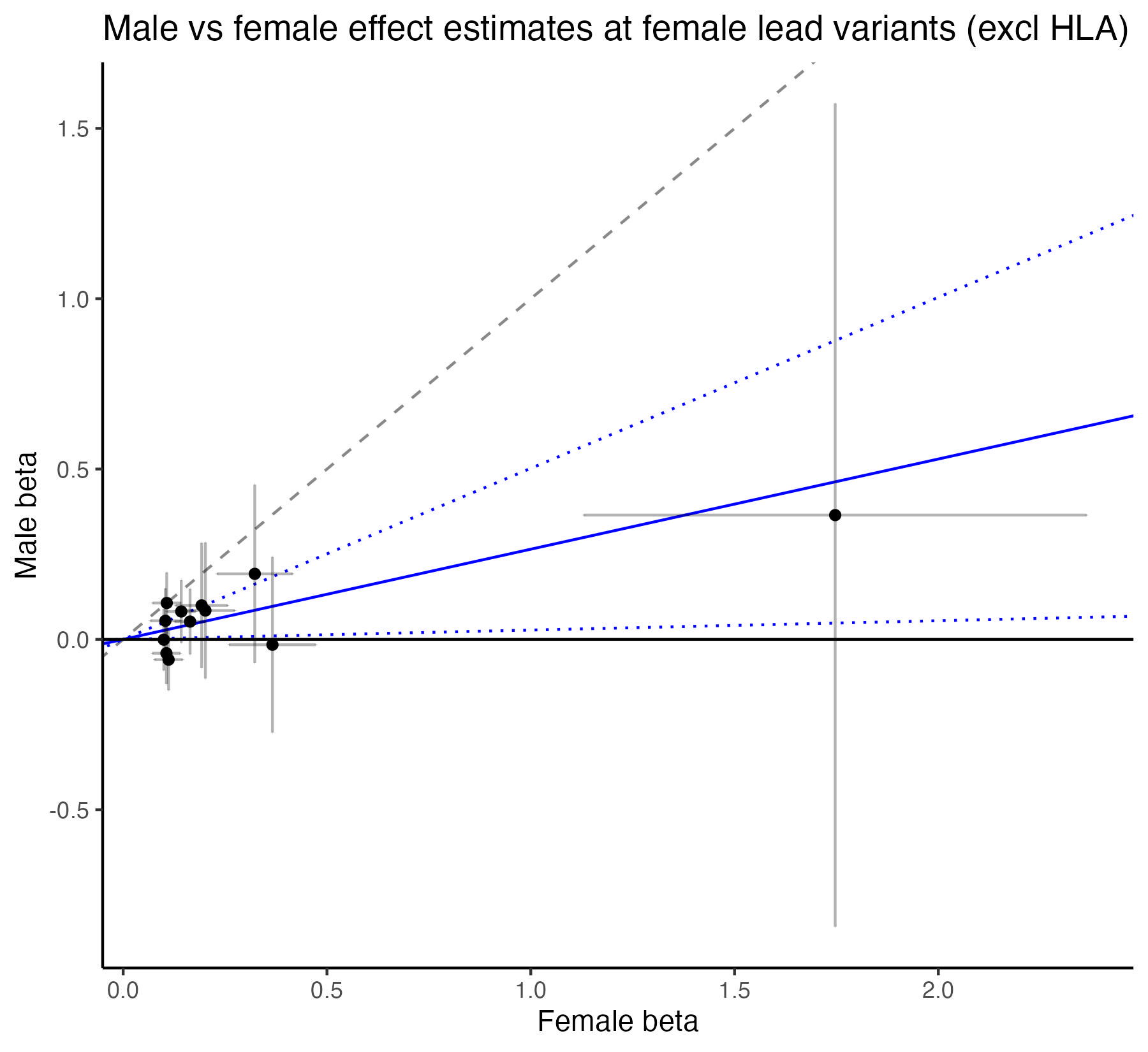** |

x-axis, effect size estimate in female LS meta-analysis; y-axis, effect size estimate in male LS meta-analysis; points, lead variants from female LS loci; error bars, 95% confidence interval for effect estimates; solid blue line, fitted inverse-variance weighted (IVW) regression slope; dotted blue lines, 95% confidence interval (CI) for IVW slope; dashed grey line, x=y. **A**, all 13 female LS loci. IVW regression slope = 0.42 [95% CI 0.21-0.64], P=9.7×10^-5^. **B**, 12 female LS loci after excluding MHC lead variant. IVW regression slope = 0.26 [95% CI 0.03-0.50], P=0.029.

**Figure S3** – Manhattan plot demonstrating evidence for association with lichen sclerosus in males and females combined


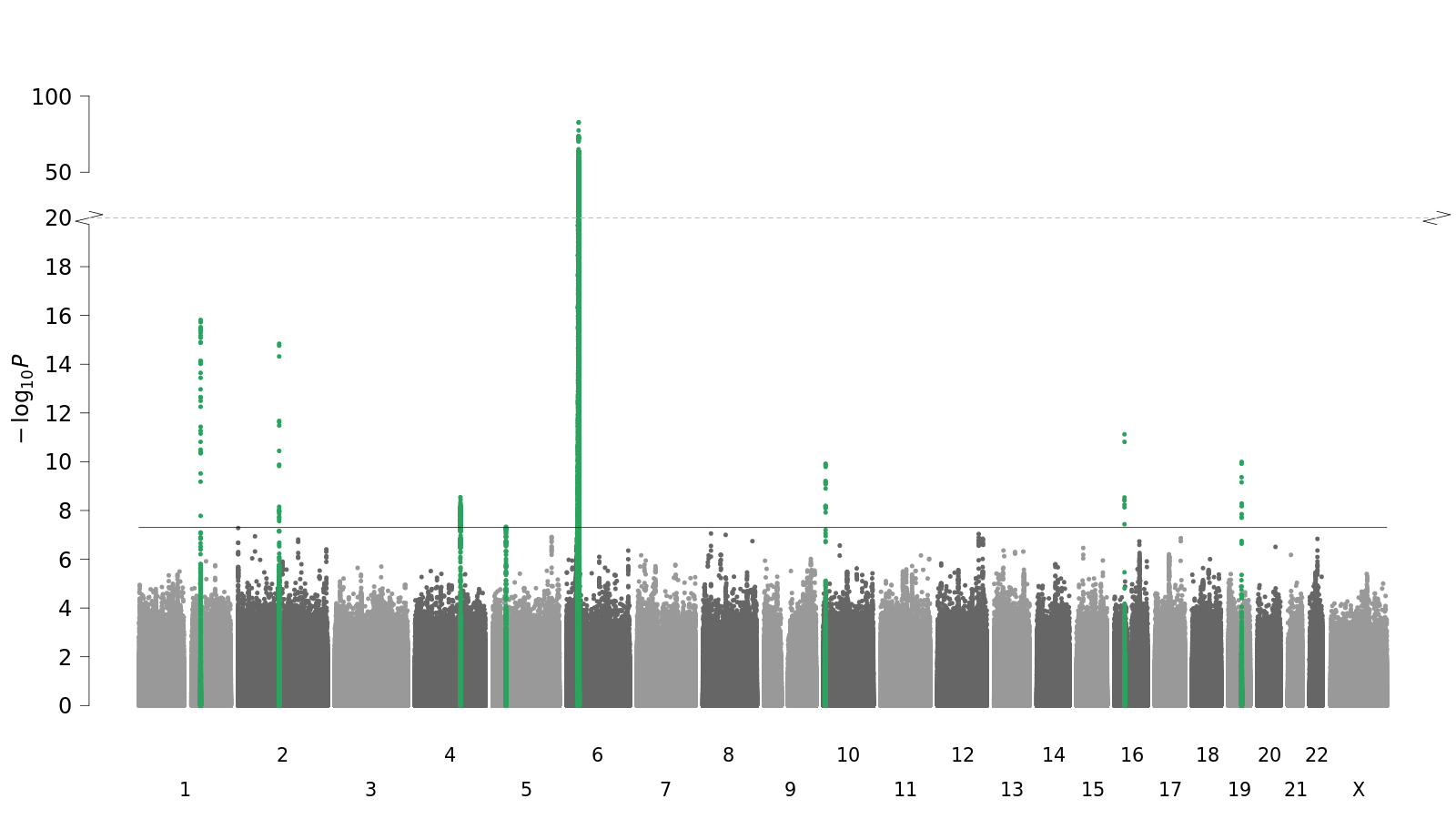


Meta-analysis of 7,651 European LS cases and 738,739 controls. x-axis, genomic position; y-axis, −log10(P-value) of association (two-sided Z-test, unadjusted for multiple tests); green points, regions associated with LS susceptibility at genome-wide significance (P = 5×10^−8^); solid horizontal line, genome-wide significance threshold; dotted horizontal line, y-axis break at 10^−20^; chromosomes (labelled 1–22 and X) are alternately shaded for clarity
