# Supplementary material for: Genome-wide meta-analysis in lichen sclerosus identifies 14 genomic risk loci
Source: Br J Dermatol. 2026 Mar 19;195(1):102–8. doi: 10.1093/bjd/ljag088 (PMC13289594; doi:10.1093/bjd/ljag088)
Supplement: ljag088_Supplementary_Data [file ljag088_supplementary_data.zip › BJD_LS_Supporting Methods.docx]

**Suppporting Methods**

**Genome-wide meta-analysis in lichen sclerosus identifies 14 genomic risk loci**

Nick Dand, Tuntas Rayinda, Eeva Silz, Laurent F Thomas, Jake R Saklatvala, Sheila M McSweeney, Chuin Ying Ung, Evangelos Christou, Fiona Lewis, Johannes Kettunen, Laura Huilaja, Ben M. Brumpton, Kristian Hveem, Mari Løset, Kaisa Tasanen, John A McGrath, Michael A Simpson, Christos Tziotzios

Correspondence: ND (nick.dand@kcl.ac.uk), MAS (michael.simpson@kcl.ac.uk) and CT (christos.tziotzios@kcl.ac.uk)

**SUPPLEMENTARY METHODS**

**Data sources and phenotype ascertainment**

This work included data from three independent European studies.

UK Biobank is a long-term observational study of 500,000 UK participants that includes data from in-person assessments and linked primary and secondary health records. UK Biobank has ethical approval from the National Health Service (NHS) National Research Ethics Service (11/NW/0382); all participants provided written informed consent. This work uses UK Biobank data under approved project 15147. Lichen sclerosus cases were identified by self-report (field 20002, code 1550), from linked hospital episode statistics (fields 41202, 41204, 41270) or death register (fields 40001, 40002) using International Classification of Diseases (10^th^ Revision) [ICD-10] code L90.0 or from linked primary care data (read v2 code M2102; read CTv3 codes M2102, X50FC, X50FE, X50FF, X50FG). Since a majority of LS cases were identified through primary care diagnoses, remaining participants without linked primary care data were excluded from the control group.

FinnGen is a Finnish biobank-based study that incorporates prospective epidemiological and disease-based cohorts and hospital biobank samples. Genetic data are linked to unique national identification numbers facilitating linkage to hospital discharge, death, and medication reimbursement registries. This study used data from FinnGen Data Freeze 12 (DF12), with lichen sclerosus cases identified using the ICD-10 code L90.0. FinnGen participants provided written informed consent for biobank research, based on the Finnish Biobank Act. Alternatively, separate research cohorts, collected prior the Finnish Biobank Act came into effect (in September 2013) and start of FinnGen (August 2017), were collected based on study-specific consents and later transferred to the Finnish biobanks after approval by Fimea (Finnish Medicines Agency), the National Supervisory Authority for Welfare and Health. Recruitment protocols followed the biobank protocols approved by Fimea. The Coordinating Ethics Committee of the Hospital District of Helsinki and Uusimaa (HUS) statement number for the FinnGen study is Nr HUS/990/2017.

The FinnGen study is approved by Finnish Institute for Health and Welfare (permit numbers: THL/2031/6.02.00/2017, THL/1101/5.05.00/2017, THL/341/6.02.00/2018, THL/2222/6.02.00/2018, THL/283/6.02.00/2019, THL/1721/5.05.00/2019 and THL/1524/5.05.00/2020), Digital and population data service agency (permit numbers: VRK43431/2017-3, VRK/6909/2018-3, VRK/4415/2019-3), the Social Insurance Institution (permit numbers: KELA 58/522/2017, KELA 131/522/2018, KELA 70/522/2019, KELA 98/522/2019, KELA 134/522/2019, KELA 138/522/2019, KELA 2/522/2020, KELA 16/522/2020), Findata permit numbers THL/2364/14.02/2020, THL/4055/14.06.00/2020, THL/3433/14.06.00/2020, THL/4432/14.06/2020, THL/5189/14.06/2020, THL/5894/14.06.00/2020, THL/6619/14.06.00/2020, THL/209/14.06.00/2021, THL/688/14.06.00/2021, THL/1284/14.06.00/2021, THL/1965/14.06.00/2021, THL/5546/14.02.00/2020, THL/2658/14.06.00/2021, THL/4235/14.06.00/2021, Statistics Finland (permit numbers: TK-53-1041-17 and TK/143/07.03.00/2020 (earlier TK-53-90-20) TK/1735/07.03.00/2021, TK/3112/07.03.00/2021) and Finnish Registry for Kidney Diseases permission/extract from the meeting minutes on 4th July 2019.

The Biobank Access Decisions for FinnGen samples and data utilized in FinnGen Data Freeze 12 include: THL Biobank BB2017_55, BB2017_111, BB2018_19, BB_2018_34, BB_2018_67, BB2018_71, BB2019_7, BB2019_8, BB2019_26, BB2020_1, BB2021_65, Finnish Red Cross Blood Service Biobank 7.12.2017, Helsinki Biobank HUS/359/2017, HUS/248/2020, HUS/430/2021 §28, §29, HUS/150/2022 §12, §13, §14, §15, §16, §17, §18, §23, §58, §59, HUS/128/2023 §18, Auria Biobank AB17-5154 and amendment #1 (August 17 2020) and amendments BB_2021-0140, BB_2021-0156 (August 26 2021, Feb 2 2022), BB_2021-0169, BB_2021-0179, BB_2021-0161, AB20-5926 and amendment #1 (April 23 2020) and it´s modifications (Sep 22 2021), BB_2022-0262, BB_2022-0256, Biobank Borealis of Northern Finland_2017_1013, 2021_5010, 2021_5010 Amendment, 2021_5018, 2021_5018 Amendment, 2021_5015, 2021_5015 Amendment, 2021_5015 Amendment_2, 2021_5023, 2021_5023 Amendment, 2021_5023 Amendment_2, 2021_5017, 2021_5017 Amendment, 2022_6001, 2022_6001 Amendment, 2022_6006 Amendment, 2022_6006 Amendment, 2022_6006 Amendment_2, BB22-0067, 2022_0262, 2022_0262 Amendment, Biobank of Eastern Finland 1186/2018 and amendment 22§/2020, 53§/2021, 13§/2022, 14§/2022, 15§/2022, 27§/2022, 28§/2022, 29§/2022, 33§/2022, 35§/2022, 36§/2022, 37§/2022, 39§/2022, 7§/2023, 32§/2023, 33§/2023, 34§/2023, 35§/2023, 36§/2023, 37§/2023, 38§/2023, 39§/2023, 40§/2023, 41§/2023, Finnish Clinical Biobank Tampere MH0004 and amendments (21.02.2020 & 06.10.2020), BB2021-0140 8§/2021, 9§/2021, §9/2022, §10/2022, §12/2022, 13§/2022, §20/2022, §21/2022, §22/2022, §23/2022, 28§/2022, 29§/2022, 30§/2022, 31§/2022, 32§/2022, 38§/2022, 40§/2022, 42§/2022, 1§/2023, Central Finland Biobank 1-2017, BB_2021-0161, BB_2021-0169, BB_2021-0179, BB_2021-0170, BB_2022-0256, BB_2022-0262, BB22-0067, Decision allowing to continue data processing until 31st Aug 2024 for projects: BB_2021-0179, BB22-0067,BB_2022-0262, BB_2021-0170, BB_2021-0164, BB_2021-0161, and BB_2021-0169, and Terveystalo Biobank STB 2018001 and amendment 25th Aug 2020, Finnish Hematological Registry and Clinical Biobank decision 18th June 2021, Arctic biobank P0844: ARC_2021_1001.

The Trøndelag Health Study (HUNT) is a population-based cohort study).^1–3^ The study was conducted at four time points over approximately 40 years (HUNT1 [1984-1986], HUNT2 [1995-1997], HUNT3 [2006-2008], and HUNT4 [2017-2019]). All inhabitants aged 20 years and over residing in Trøndelag County, Norway, were invited to participate. The surveys included clinical measurements, blood sampling, and questionnaires covering general health measures and a broad range of self-reported diseases and symptoms. Participants were also linked to regional and national health registries through their unique national identification number. Participation in HUNT is based on informed consent, and the study has been approved by the Norwegian Data Protection Authority and the Regional Committee for Medical and Health Research Ethics in Central Norway (REK Reference number 27420). Lichen sclerosus cases were identified using the ICD-10 code L90.0, as recorded in hospital and private specialist records, including those from dermatologists. Control data excluded participants with ICD-10 L90.0, and ICD-9 codes 697.8 and 697.9 (unspecified lichen).

**Genetic data and association testing**

Whole-genome imputed genotype data were obtained from UK Biobank along with quality control (QC) metrics derived by the UK Biobank central team.^4^ Briefly, these comprised genotype array data generated using the Affymetrix UK BiLEVE and UK Biobank Axiom arrays, imputed using IMPUTE2 software against a reference panel comprising UK10K haplotypes and 1000 Genomes Phase 3 samples. We excluded samples whose QC metrics indicated that they exhibited sex mismatch, excess relatedness or heterozygosity, missingness greater than 5% across all markers, missingness greater than 2% across well-called (>90%) markers, or were not of homogenous ancestry (to limit potential bias due to population stratification; remaining samples were from the white British ancestry group). We performed a genome-wide association study using imputed variants with an imputation r^2^ >0.7 and minor allele frequency >0.1%. Association testing was performed using a logistic mixed model, accounting for birth year, genotyping array and first 5 ancestry principal components as covariates, plus sex for the sex-combined analysis, using REGENIE v3.2.4.^5^ Classical allele association testing was performed on a subset of unrelated samples (females: 2,148 cases, 83,554 controls; males: 241 cases, 72,365 controls) in PLINK v1.9^6^ based on four-digit HLA alleles imputed by the UK Biobank central team, and using a logistic regression model adjusted for genotyping array and the first 20 ancestry principal components.

FinnGen genotypes were generated using Affymetrix and Illumina arrays, with QC and whole-genome imputation against a population-specific Sequencing Initiative Suomi (SISu) v3 reference panel, as described elsewhere.^7^ In females, males and combined sexes, association testing was performed for variants with a minor allele count ≥5 using a logistic mixed model approach (REGENIE v2.2.4), adjusting for age, the top 10 principal components and genotyping batch, plus sex for the combined-sex analysis. Results were lifted over from GRCh38 to GRCh37 positions for harmonization with UK Biobank and HUNT results, using the UCSC Liftover tool.^8^ Final numbers for cases, controls and tested variants are given in Supplementary Table 1. Association testing for four-digit classical HLA alleles, imputed using HIBAG R library^9^ and imputation models trained on Finnish individuals^10^ was performed using logistic regression implemented in PLINK2 v2.00a6LM, and included a subset of unrelated individuals (females: 1,906 cases, 155,749 controls; males: 303 cases, 120,802 controls).

In HUNT, whole-genome imputed data were derived from Illumina HumanCoreExome array genotypes based on a reference panel comprising Haplotype Reference Consortium samples, as described previously.^11^ All participants included in the final analysis were of homogeneous European ancestry. Association testing on variants with minor allele count ≥10 (per analysis) and imputation INFO score >0.3 was performed using SAIGE version 1.0.3^12^, including birth year, genotyping batch and the first 10 ancestry principal components as covariates, plus sex for the combined-sex analysis. Final numbers for cases, controls and tested variants are given in Table S1. Classical HLA alleles are not available for HUNT participants.

**Meta-analysis**

Standard error-weighted meta-analysis was performed separately for male, female and combined-sex GWAS using METAL v2020-05-05.^13^ Susceptibility loci were determined by the identification of lead variants: those variants exhibiting genome-wide significant association (meta-analysis p-value <5.0×10⁻⁸) and being at least 500 kb from a variant with a lower p-value. Due to long-range linkage disequilibrium (LD), the MHC region on chromosome 6 (25.4–33.4 Mb) was considered a single susceptibility locus, and further interrogated via association testing of imputed classical HLA alleles. Similarly, closer inspection of the chromosome 4q27 locus showed an extended pattern of LD and as such the locus definition was extended to ±1000 kb.

To estimate the liability-scale common SNP heritability for lichen sclerosus for a range of population prevalences (0.5%, 1%, 2%), we used LD score regression (LDSC) with precomputed 1000 Genomes LD scores.^14^

To compare estimated effect sizes between females and males, we took the lead variants for the 13 female LS susceptibility loci and looked up effect size estimates and standard errors in the male meta-analysis results. We performed inverse-variance weighted regression using the ‘MendelianRandomization’ R package (v 0.10.0).^15^

**Fine-mapping**

For non-MHC loci, the ‘coloc’ R package (v5.2.3)^16^ was used with default settings to calculate approximate Bayes factors for each variant, and derive posterior probabilities (PP) that each variant causes the observed association signal (under the assumption of a single causal variant). Variants up to ±500 kb from the lead variant were included, except for the 4q27 locus where ±1000 kb was used (as described above). We constructed 95% credible sets as the minimum set of variants whose cumulative posterior probability exceeded 0.95. Other than the chromosome 17q24.3 locus, which failed to resolve to a credible set, all 95% credible set variants were annotated using ANNOVAR (via the wANNOVAR interface (2024 version))^17^ to identify variants in protein coding regions.

**Establishing mechanisms via eQTL and phenome-wide variant lookup**

To determine potential candidate genes for which there is evidence that LS susceptibility variants could be acting via an effect on gene expression, we: (i) identified a subset of ‘strong candidate’ susceptibility variants, (ii) searched the eQTL Catalogue^18^ for reported associations between these variants and expression levels in a relevant tissue, and (iii) formally tested for colocalization between LS susceptibility and eQTL signals. Specifically:

(i) Strong candidate variants were defined as those in non-MHC loci having a fine-mapping posterior probability of being causal ≥ 0.1. We identified 27 in total across female loci; none were found for the combined-sex chromosome 5p13.2 locus (max. PP 0.067).

(ii) eQTL Catalogue lookup was performed using the eQTL Catalogue RESTful API (https://www.ebi.ac.uk/eqtl/api/docs). We limited our search to eQTLs reported in skin- and immune-related tissues, namely: "B cell", "blood", "CD16+ monocyte", "CD4+ CTL cell", "CD4+ memory T cell", "CD4+ T cell", "CD4+ TCM cell", "CD4+ TEM cell", "CD56+ NK cell", "CD8+ T cell", "CD8+ TCM cell", "CD8+ TEM cell", "dendritic cell", "dnT cell", "gdT cell", "macrophage", "MAIT cell", "memory B cell", "monocyte", "neutrophil", "NK cell", "plasma", "plasmacytoid dendritic cell", "platelet", "skin", "skin (suprapubic)", "T cell", "Tfh cell", "Th1 cell", "Th17 cell", "Th2 cell", "thyroid", "Treg memory", "Treg naive", "vagina". We further filtered our results for eQTLs with genome-wide significant p-values (<5.0×10⁻⁸).

(iii) For each gene with an eQTL identified, we selected the tissue and study with the strongest eQTL evidence to test for colocalization with LS association based on variants tested in both studies. We downloaded region-wide eQTL summary statistics and performed Bayesian colocalization analysis using the ‘coloc’ R package (v5.2.3) based on a predefined prior probability of colocalization of 10⁻⁵. Where colocalization was not supported (posterior probability of model H_4_ [“both traits are associated and share a single causal variant”] <0.5), we tested for colocalization with a second eQTL dataset for the same gene (if available). We stopped after two eQTL datasets if positive evidence for colocalization had implicated other likely causal genes at the same locus.

We also looked up strong candidate variants in GWAS Catalog^19^ using the R package ‘ggrapid’ ^20^, performing a similar colocalization exercise for selected traits of interest.

**SUPPLEMENTARY REFERENCES**

1 Næss M, Kvaløy K, Sørgjerd EP, *et al.* Data Resource Profile: The HUNT Biobank. *Int J Epidemiol* 2024; **53**. doi:10.1093/ije/dyae073.

2 Åsvold BO, Langhammer A, Rehn TA, *et al.* Cohort Profile Update: The HUNT Study, Norway. *Int J Epidemiol* 2023; **52**:e80–91.

3 Krokstad S, Langhammer A, Hveem K, *et al.* Cohort Profile: the HUNT Study, Norway. *Int J Epidemiol* 2013; **42**:968–77.

4 Bycroft C, Freeman C, Petkova D, *et al.* The UK Biobank resource with deep phenotyping and genomic data. *Nature* 2018; **562**:203–9.

5 Mbatchou J, Barnard L, Backman J, *et al.* Computationally efficient whole-genome regression for quantitative and binary traits. *Nat Genet* 2021; **53**:1097–103.

6 Chang CC, Chow CC, Tellier LCAM, *et al.* Second-generation PLINK: rising to the challenge of larger and richer datasets. *Gigascience* 2015; **4**:s13742-015-0047–8.

7 Kurki MI, Karjalainen J, Palta P, *et al.* FinnGen provides genetic insights from a well-phenotyped isolated population. *Nature* 2023; **613**:508–18.

8 Hinrichs AS, Karolchik D, Baertsch R, *et al.* The UCSC Genome Browser Database: update 2006. *Nucleic Acids Res* 2006; **34**:D590-8.

9 Zheng X, Shen J, Cox C, *et al.* HIBAG--HLA genotype imputation with attribute bagging. *Pharmacogenomics J* 2014; **14**:192–200.

10 Ritari J, Hyvärinen K, Clancy J, *et al.* Increasing accuracy of HLA imputation by a population-specific reference panel in a FinnGen biobank cohort. *NAR Genomics Bioinforma* 2020; **2**:lqaa030.

11 Brumpton BM, Graham S, Surakka I, *et al.* The HUNT study: A population-based cohort for genetic research. *Cell genomics* 2022; **2**:100193.

12 Zhou W, Nielsen JB, Fritsche LG, *et al.* Efficiently controlling for case-control imbalance and sample relatedness in large-scale genetic association studies. *Nat Genet* 2018; **50**:1335–41.

13 Willer CJ, Li Y, Abecasis GR. METAL: fast and efficient meta-analysis of genomewide association scans. *Bioinformatics* 2010; **26**:2190–1.

14 Bulik-Sullivan BK, Loh P-R, Finucane HK, *et al.* LD Score regression distinguishes confounding from polygenicity in genome-wide association studies. *Nat Genet* 2015; **47**:291–5.

15 Patel A, Ye T, Xue H, *et al.* MendelianRandomization v0.9.0: updates to an R package for performing Mendelian randomization analyses using summarized data. *Wellcome open Res* 2023; **8**:449.

16 Giambartolomei C, Vukcevic D, Schadt EE, *et al.* Bayesian test for colocalisation between pairs of genetic association studies using summary statistics. *PLoS Genet* 2014; **10**:e1004383.

17 Wang K, Li M, Hakonarson H. ANNOVAR: functional annotation of genetic variants from high-throughput sequencing data. *Nucleic Acids Res* 2010; **38**:e164.

18 Kerimov N, Tambets R, Hayhurst JD, *et al.* eQTL Catalogue 2023: New datasets, X chromosome QTLs, and improved detection and visualisation of transcript-level QTLs. *PLoS Genet* 2023; **19**:e1010932.

19 Cerezo M, Sollis E, Ji Y, *et al.* The NHGRI-EBI GWAS Catalog: standards for reusability, sustainability and diversity. *Nucleic Acids Res* 2025; **53**:D998–1005.

20 Magno R, Maia A-T. gwasrapidd: an R package to query, download and wrangle GWAS catalog data. *Bioinformatics* 2020; **36**:649–50.
